# Self-Sealing Cementitious Materials by Using Water-Swelling Rubber Particles

**DOI:** 10.3390/ma10080979

**Published:** 2017-08-22

**Authors:** Leyang Lv, Erik Schlangen, Feng Xing

**Affiliations:** 1 Micromechanics Laboratory (MICROLAB), Faculty of Civil Engineering and Geosciences, Delft University of Technology, Stevinweg 1, 2628 CN Delft, The Netherlands; Erik.Schlangen@tudelft.nl; 2 Guangdong Province Key Laboratory of Durability for Marine Civil Engineering, School of Civil Engineering, Shenzhen University, Shenzhen 518060, China; xingf@szu.edu.cn

**Keywords:** water swelling rubber, self-sealing, cementitious materials, bridging effect

## Abstract

Water ingress into cracked concrete structures is a serious problem, as it can cause leakage and reinforcement corrosion and thus reduce functionality and safety of the structures. In this study, the application of water-swelling rubber particles for providing the cracked concrete a self-sealing function was developed. The feasibility of applying water-swelling rubber particles and the influence of incorporating water-swelling rubber particles on the mechanical properties of concrete was investigated. The self-sealing efficiency of water-swelling rubber particles with different content and particle size was quantified through a permeability test. The sealing effect of the water swelling rubber particles was monitored by X-ray computed tomography. The experimental results show that, by using 6% of these water swelling rubber particles as a replacement of aggregates in concrete, up to 64% and 61% decrease of water permeability was realized for 0.7 mm and 1.0 mm cracks. Furthermore, when the concrete cracks, the water swelling rubber particles can act as a crack bridging filler, preventing the crack from fully separating the specimens in two pieces.

## 1. Introduction

Cracking related deterioration seriously influences the integrity and durability of concrete. Cracks cause water leakage, therefore reducing the functionality of civil structures such as dams, retaining walls, tunnels. Cracks also induce aggressive agents by water transport through the crack, which is often detrimental to the service life of reinforced concrete structures. Furthermore, when the ingress of aggressive agents leads to corrosion of the reinforcement, it can even pose a threat to the safety of the structure. Although better structural design and content proportion of components can help to decrease the probability of cracking, cracks, as an unavoidable feature of concrete, need a more efficient and automatic way to reduce the hazardous effect it brings.

In recent years, inspired from biological self-healing phenomenon, the concept of self-sealing concrete began to show its beneficial and great application potential in the field of civil engineering [1,2,3]. Up to now, several solutions were studied targeting to reduce the permeability of concrete automatically. Among these, autogenous self-healing is one of the main promising mechanisms in cementitious materials. This oldest self-healing mechanism has already been observed in concrete structures that can be tracked back to the nineteenth century [4]. The advantage of this method is that no additives are needed to mix into concrete. Therefore, the chemical and mechanical properties of concrete will not be influenced. When the cracked concrete is exposed to water, the cracks may autogenous heal under a certain environmental condition. However, the limitations are also obvious. The autogenous self-healing can only occurs for a narrow crack with crack width typically less than 0.3 mm [5]. In addition, there are several specific requirements to the healing conditions such as temperature, wet/dry cycle and the ions in the water which cannot be easily achieved in practice. Autonomic healing is another self-healing mechanism that is often applied to compensate the drawbacks of autogenous self-healing in cementitious materials. By mixing healing agent such as bacteria, expansive powder mineral, superabsorbent polymer etc., the cracks can be healed more efficiently, whenever the healing agent is triggered upon crack formation or other stimuli. The microcapsule based self-healing concept has shown its application potential in cementitious materials [3,6,7,8,9,10]. While the healing effect can be hindered due to the weak bonding strength between the polymeric microcaspules and inorganic cementitious matrix. The feasibility of using Bacterial spores as healing agent has been also found and developed [2,3,5,11]. However, similar to autogenous self-healing, the efficiency of bacteria-based self-healing is restricted to the duration of immersion and the composition of the incubation solution, which brings a challenge in real situations. More recently, water-swelling materials such as superabsorbent polymer (SAP) were found to be a new type of self-healing materials that holds a potential in crack self-sealing [12,13,14,15]. The result shows that the application of SAP can provide up to 85% and 98% decrease of the peak flow and cumulative flow, leading to the complete sealing of a 0.3 mm crack. However, practical application of SAP in civil engineering structures was rather limited due to its detrimental effect on compressive strength. Recent results show that the addition of 5% SAP by weight of cement will lead to a reduction of 80% of compressive strength of concrete [12].

To find a material which is practically applicable of providing a long-term sealing capacity of concrete structure, three requirements should be satisfied: (1) the materials can survive in and have a good bonding with concrete structure; (2) the sealing function of the selected material can be triggered automatically upon water ingress or other stimuli without or with less human intervention and (3) the incorporation of the material should have only limited negative effect on the mechanical properties of concrete structure, and if possible, even enhance it. Previously, the possibility of applying rubber phases as partial replacement of sand in concrete has been investigated. This method attempted to make the concrete, a traditional building material, embrace more versatile performances such as higher energy dissipation, ductility, durability, damping ratio, impact resistance and toughness [16,17,18,19]. The results of these studies show that the waste tire rubber reinforced concrete (RRC) could be an ideal ecological component of concrete when it was applied as a replacement of aggregate and subjected to dynamic loading conditions. While, to our knowledge, no research can be found on the application of rubber with water swelling function to provide the concrete with a combined self-sealing and self-bridge function. Water swelling rubber is a new type of construction material which has been widely used for sealing of precast concrete elements (e.g., man holes), shaft rings and pipe lead-throughs etc. The matrix of water swelling rubber is butyl rubber, a copolymer of isobutylene with isoprene, also known as Isoprene Isobutylene Rubber (IIR). The expanding property results from the irrevocable bonding of polyurethane-based water-expanding polymer resin which has been pre-mixed in the IIR during the fabrication process. The physical characteristics of water swelling rubber is similar to the ordinary IIR. While, comparing with SAP, the benefit of this type rubber is, only limited volume of can swell up within 24 h. This 24 h swelling delay provide a precious time for the mortar to be harden. The reason for this delay can be attribute to the IIR rubber prolonged the time for those pre-mixed polyurethane-based water-expanding polymer resin in it from being swell by the moisture in the water. After 7 days on contact with water, the water swelling rubber can finally swell up to 250% of its original volume. The benefit of this rubber is, there will be a swelling delay when it first contact with water. Meanwhile, due to the sticky texture and strong surface bonding strength of water swelling rubber, it is expected that the expanded rubber could have a good adhesion to the crack faces, provide some crack bridging effect and keep the cracked pieces together.

Accordingly, the core concept of this work is to utilize water swelling rubber particles (WSRPs) as a new ingredient of cementitious materials to enable the ability for the cracked concrete structure to reduce its permeability and partially regain its deteriorated mechanical properties. Once cracks have formed in the concrete structure and water penetrates, the rubber may swell gradually, expand along the crack and partly seal the crack. Subsequently, the embedded rubber can also act as reinforcing particles that provide an extra bond strength and prolong the serviceability of cracked concrete. In this study, the feasibility of applying WSRPs in crack self-sealing was proven by both a simulated crack and X-ray Computed Tomography (XCT) technology. The influence of incorporation of WSRPs on the mechanical properties was investigated. Water permeability tests were carried on a series of WSRPs embedded mortar specimens to study the influence of WSRPs size and content, and crack width on the self-sealing effect. Finally, the capability of crack bridging to the cracked mortar, provided by the embedded WSRPs, was characterized.

## 2. Experimental

### 2.1. Materials

**Figure 1:**
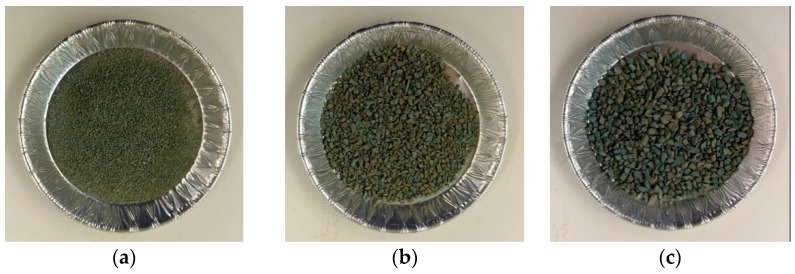
Images of WSRP with size range of (**a**) 0.5–1 mm (S); (**b**) 1–2 mm (M) and (**c**) 2–4 mm (L).

Water swelling rubber (AQUA TACKSEAL) was provided by TPH Bausysteme GmbH, Norderstedt, Germany. To obtain the water swelling rubber particles (WSRPs), the as received materials were first frozen by liquid nitrogen and then crushed immediately in a grinder by hand. Before the mass water swelling rubber began to be crushed into smaller particles, the cement powder will be added in the grinder. These cement powder will be act as desiccants, sticking on the new formed surface of WSRPs to prevent the new formed particles from being stick together. After that, the crushed WSRPs were sieved by a screen with mesh diameter of 0.5 mm, 1 mm, 2 mm and 4 mm respectively. Figure 1 shows the images of granular WSRPs used in this study highlighting the difference in particle size, (a) S size (b) M size and (c) L size.

Ordinary Portland cement, CEM I 52.5 N and CEN standard RILEM sand was used for the preparation of mortar samples. The grading of the sand is given in Table 1, which complies with the requirements of EN 196-1 (§ 5) and ISO 679: 2009 (§ 5). Deionised water was used as batch water.

### 2.2. Sample Preparation

**Figure 2:**
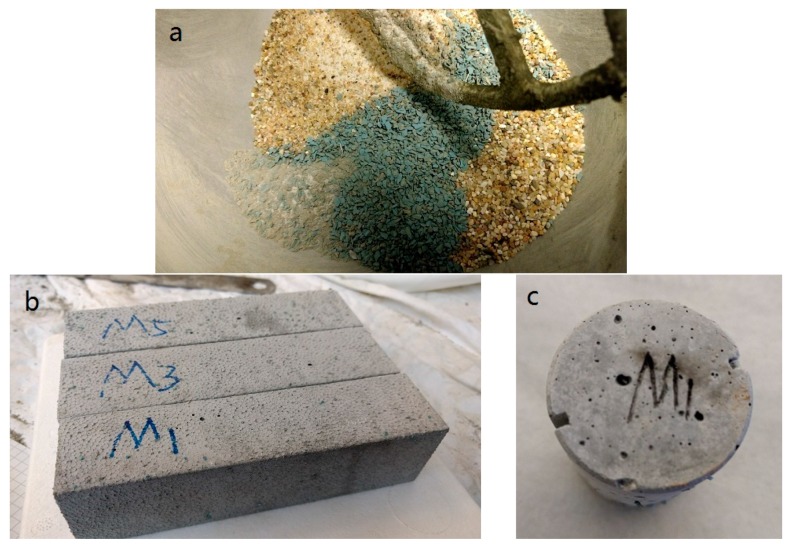
Images of (**a**) the mixture prepared for mixing procedure; (**b**) prisms for mechanical test and (**c**) cylinder with double notches for water permeability test.

According to the EU standard EN 196-1 [20], the amount of cement was kept constant at 450 g, water to cement ratio was selected as 0.5, and 1350 g filler (Rilem sand + WSRPs) was used as fine aggregate. Mortar was chosen as a representative material for concrete. The mixture contents of cement mortar are shown in Table 2. Four series of specimens including S, M, L, and B were made. For those WSRPs incorporated cement mortar, series S, M and L represent the size of incorporated WSRPs, ranging 0.5–1 mm, 1–2 mm and 2–4 mm. The following numbers 1, 3 and 6 stand for the volume ratio of which the sand were replaced by WSRPs. Series B, as reference, is pure mortar without WSRPs. To avoid any interference and complication in the results, superplasticizer was not used. Mortar mixtures were mixed in a Hobart mixer by a certain order. In the first step, cement, sand and WSRPs were put in the mixer and mixed thoroughly at low gear (60 rpm) for 60 s. Water was then added in the mixer and mixed at low gear (60 rpm) for another 60 s. After the mixing, the mortar mixtures were cast into a standard prismatic moulds (40 × 40 × 160 mm^3^) and cylinder moulds (60 mm long and with a diameter of 33.5 mm). Following the work of Palin et al. [21], the cylinder mould has two opposite notches on both long side of the height (2 mm wide and 3 mm deep). The casted moulds were then vibrated on a shaking table to remove the voids. All the specimens were de-moulded after 24 h and then cured in a fog room at normal curing condition (20 °C, >95% RH) for 28 days. For each mix (with different ID), 6 standard prisms and 9 cylinders were casted. Figure 2 illustrates images of the mixture prepared for mixing procedure (a); the prisms for mechanical test (b) and the cylinder with notches for water permeability test (c).

### 2.3. Morphology of WSRPs in Mortar

The morphology of cement mortar containing L and M sized WSRPs was imaged by a digital camera. The size of specimens that was used for the observations is a 40 × 40 × 5 mm^3^ slice. The microstructure of the WSRPs embedded mortar was characterized by an environmental scanning electron microscope (ESEM) under low vacuum mode (XL30, Philips, Amsterdam, The Netherlands). All imaging was performed in low vacuum. Before the test, the surface of the sample is ground by four different grinding papers (500#, 800#, 1200#, 4000#). Then, the sample was polished by hand on a lapping table using diamond paste with particle diameter of 6 μm, 3 μm, 1 μm and 0.25 μm. The polishing time is 5 min for each step.

### 2.4. Feasibility of Applying WSRPs in Concrete—Proof of Concept 

#### 2.4.1. Swelling Effect of WSRPs in Simulated Crack

The swelling effect of WSRPs in concrete was first investigated using a simulated crack. To clearly see the swelling effect, a glass sheet was used to cover the cross section of WSRPs containing mortar sample. In order to ensure the crack width, a double sided plastic tape with a thickness of 0.8 mm was inserted in between the glass sheet and the section surface of the sample to control the simulated crack width to 0.8 mm. The swelling effect of the WSRPs was recorded by acquiring images with a digital camera after 0, 3, 7 days of immersion in deionized water.

#### 2.4.2. Sealing Effect of WSRPs in Mortar

X-ray computed tomography (XCT) is a versatile, non-destructive inspection method, which has been widely applied in various fields of research [22,23]. Recently, the application of this technique has also been extended to the area of civil engineering [10,24,25,26]. As a non-destructive imaging technique, XCT provides an approach to study the internal information of a cementitious structure based on the principle of different X-ray absorption between phases or elements [27]. In this study, X-ray computed tomography (XCT) scanning technology (Nanotom, GE Inspection Technologies, Lewistown, PA, USA) was applied to proof the swelling and sealing function of WSRPs within hardened mortar. To visualize the sealing effect of WSRPs in mortar, the WSRPs embedded mortar cylinder before and after the sealing process was subjected to XCT. In between, the cylinder was immersed in deionised water for 7 days. In this study, the raw XCT images were acquired at an acceleration voltage of 120 kV with an exposure time of 4 s and X-ray power of 8 W. The resolution of CT scans was set to 20 μm. The final data set of XCT consisted of 1440 radiographs of which each image was acquired with a 0.25° rotation. Then, phase retrieval and tomographic reconstruction were performed to improve the boundaries and signals using the software supplied by the manufacturer. A series of reconstructed tomographic images (X–Z plane) were consequently imported into a commercial software (VGStudio MAX 3.0, Volume Graphics GmbH, Heidelberg, Germany) for segmentation and 3D visualization. Section images of the reconstructed 3D volume containing the information of the crack and WSRPs were compared. The crack self-sealing effect was quantified via an additional analysis of the 3D volume by comparing the volume fraction of WSRPs and crack in regions around the healed crack, before and after the healing process.

### 2.5. Influence of the WSRPs on the Mechanical Properties of Mortar 

The flexural and compression strength of the WSRPs embedded cement mortar was measured according to TS EN 1015-11 (2000) [28]. In order to obtain the flexural strength of the mortars, 40 × 40 × 160 mm^3^ specimens were used. The specimens were tested after 28 days curing for flexural strength under three-point loading with the span between supports being of 100 mm. The average of results obtained from three prismatic specimens was reported as flexural strength. The two broken parts of the 40 × 40 × 160 mm^3^ retained after the flexural strength test were used for compressive strength. The loading rate was 500 N/s. The loading area was 40 × 40 mm^2^. The average of results obtained from six broken pieces was reported as compressive strength. To investigate the influence of ageing on the mechanical properties, same test was conducted again on the remaining three specimens which were stored in lab environment for 28 days after the first 28 days of hydration.

### 2.6. Evaluation of Self-Sealing Ability

#### 2.6.1. Crack Calibration

A crack with a certain width was induced on the cylinder specimens for the evaluation of the water sealing effect. Before the test, two steel rods were placed at each side of the notch. Then an Instron 8872 servohydraulic testing machine (Instron Corp., Canton, MA, USA) was used to apply a compressive load on the steel rods until the cylinder split diametrically. Spacers with a width of 2.4, 2.7 and 3.0 mm were placed thoroughly in the notches. Since the width of notches is 2 mm, therefore by placing spacers with a width of 2.4, 2.7 and 3.0 mm, cracks widths of 0.4, 0.7 and 1.0 mm can be achieved. Then the spacers were moved to half way of the notches, and a two-component adhesive, Plex 7742 and Pleximon 801 (Evonik Rohm GmbH, Darmstadt, Germany), was mixed and applied on the space of notches. After the adhesive was harden, the spacers were removed and the rest space of notches were completely filled by adhesive. A watertight permeability cell was used to seal the cylinder specimens and to connect them with the permeability test setup. To prevent the water leakage from pores or defects of cylinders during the permeability test, before the cylinder was placed into the cell, rubber rings were attached on both ends of the cylinder specimen. In the end, stereomicroscope analysis was applied to measure the actual widths of cracks. A tolerance variation was set for those sample with a crack variable over a certain criteria to be screened out from being permeability test. For the 0.4 mm sample, the tolerable variation is 0.05 mm, for 0.7 mm sample, the tolerable variation is 0.07 and for 1.0 mm sample, the tolerable variation is 0.1 mm. This means the variable of those tested sample within the tolerable variation was regarded as and reflected on the standard deviation of water permeability.

#### 2.6.2. Water Permeability

The water permeability was tested according to the work of Palin et al. [29]. Briefly, the permeability cells were first attached to the bottom of permeability setup. Water was then poured into reservoirs at the top of each setup. To provide an almost constant water pressure of 0.1 bar, the water level of each permeability setup was manually controlled to a height of 1 m from the top of the water surface to the bottom of the permeability cell. To start the test, taps of the reservoirs were released. Water flowing through the cracks and was collected separately by buckets beneath the testing setups. The weight of the water in the buckets was weighted and recorded two times at two continuous 5 min. The permeability test was performed after 0 and 7 days submersion of the crack-induced specimens in tap water. The permeability (cm^3^·s^−1^) is simply defined as the volume of flowed out water from the crack (cm^3^) divides the recorded time (s). For each data point, 3 replicate samples were tested.

### 2.7. Visual Assessment of Crack Bridging Function of WSRPs

The crack bridging function is a unique characteristic of WSRPs which can prevent the crack from fully separating the specimens in two pieces. To visualize the function, a mortar prism with WSRPs embedded was fractured by tensile force using Axial Tension-Compression Systems (8872, Instron Corp., Canton, MA, USA). Then the tensile force was removed, the cracked area of WSRPs embedded mortar was recorded by a digital camera.

## 3. Results and Discussion

### 3.1. Morphology of WSRP in Mortar

**Figure 3:**
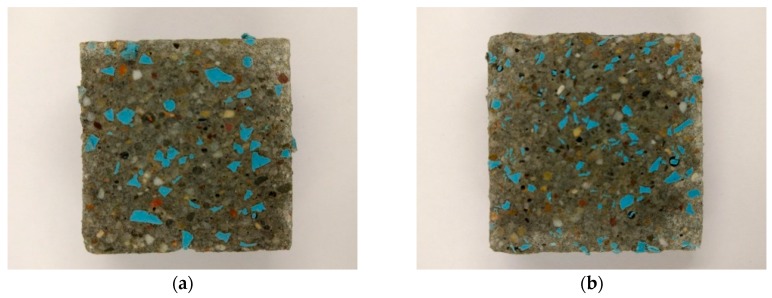
Images of the cross section of mortar specimen with WSRPs embedded (**a**) L series (particle size: 2–4 mm); (**b**) M series (particle size: 1–2 mm). The length of scale bar in images is 10 mm.

**Figure 4:**
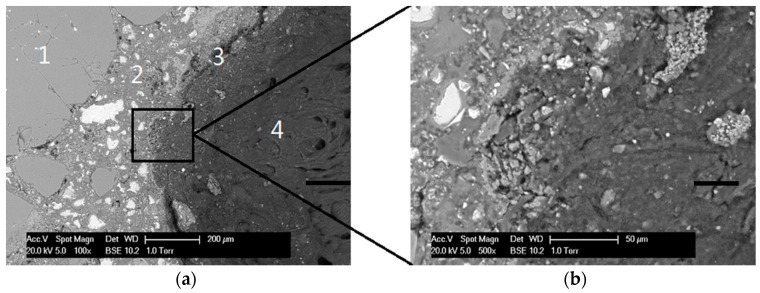
ESEM images showing the microstructure of interface zone between WSRP and mortar matrix at the age of 28 days. (**a**) at left side highlights (1) the aggregate particles, (2) hydrated and unhydrated cement paste, (3) WSRPs and cement paste interface and (4) embedded WSRPs; (**b**) at the right side shows an enlarged view of the microstructure of the interface.

The morphology of WSRPs embedded mortar is shown in Figure 3, highlighting the dispersibility, size and shape of the embedded WSRPs. As can be seen from the figure that, both large size and medium size WSRPs (blue) are well dispersed in mortar. No obvious voids can be found around the WSRP, demonstrating that the quartz shape WSRPs have a good connection with the mortar matrix, reminiscent of aggregates. For better understanding the influence of WSRPs on the surrounding microstructure of mortar. The interface zone between WSRPs and mortar matrix at the age of 28 days of the sample was investigated by ESEM. It can be clearly seen from Figure 4 that no macro or micro-cracks were found due to the WSRP. Meanwhile, the magnified image in Figure 4b tends to show that WSRPs has an effective adhesion with cement paste. All the above-mentioned phenomena suggest that the embedded WSRPs do not change evidently during the process of hydration and the existence of WSRPs will not influence the microstructure of the surrounding mortar.

### 3.2. Proof of Concept

#### 3.2.1. Swelling Effect of WSRPs at Simulated Crack

**Figure 5:**
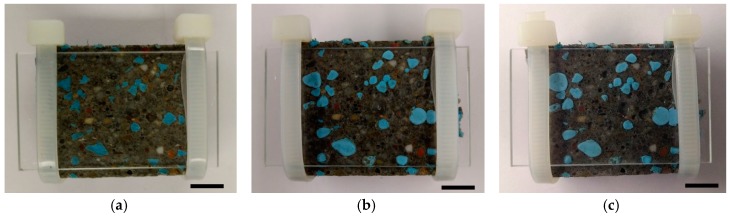
Swelling effect of WSRPs in a simulated crack with a crack width of 0.8 mm at (**a**) 0 day; (**b**) 3 days and (**c**) 7 days of the specimens being immersed in water. The length of scale bar in images is 10 mm.

The self-sealing function of the WSRPs in cementitious materials is realized through swelling of WSRPs and expanding into the cracks. To proof the feasibility of applying WSRPs in mortar to reduce the volume of crack and further decrease the permeability, a preliminary test was carried out. In order to have a direct view of the swelling process of WSRPs, a glass sheet was used as one side of the crack, covering on a slice of WSRPs embedded mortar. Figure 5 shows the swelling process of WSRP in the simulated crack. As can be seen from the figure, at the initial stage (0 days), the WSRPs (blue) keep their original shape. After 3 day of immersion in water, the embedded WSRPs begin to swell and expand in the crack space in which some of them join together which partly blocks the crack space. As the immersion time goes to 7 days, the WSRPs expand only a little bit. The results demonstrate that the self-sealing action of WSRPs can be activated and reach its maximum function within 7 days.

#### 3.2.2. Self-Sealing Effect of WSRPs in Mortar 

**Figure 6:**
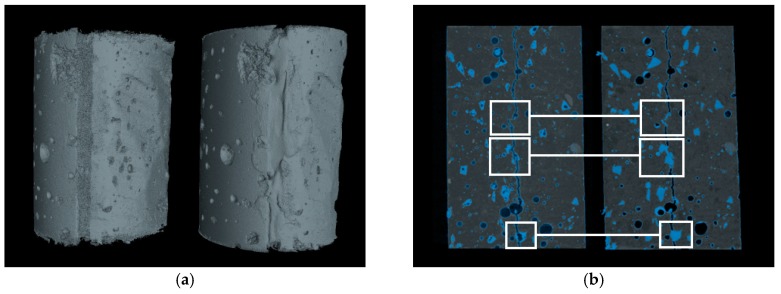
Reconstructed XCT images of (**a**) samples with WSRPs embedded cement mortar and (**b**) samples before and after sealing of the crack by WSRPs (blue particles). The length of scale bar in images is 10 mm.

In this study, XCT was used to study the self-sealing effect of WSRPs on cracks in the hardened mortar specimens. In the first step, the reconstructed 3D volumes of the cracked mortar samples, before and after the water healing process, were coupled by selecting the same rotation angle (Figure 6a). In the second step, tomographic slices of a longitudinal cross-section of the reconstructed 3D volumes were selected and compared in detail (Figure 6b). Finally, various phases of composite material is differentiated and colored base on the principle of different grey value (result from different density of material) under the X-ray. Therefore, only those material with the same density can be selected out. Since the rubber is basically a polymer which has much less density than cementitious material, all grey value fall into a certain band of grey value chart should be the material of rubber. So the material within this grey value was selected and then labeled with a blue color. It can be clearly seen that, before the water healing process, the crack runs through the entire specimen. This means that the water can permeate through the crack directly without any obstacle. While, after the healing process, WSRPs (blue) which are located along the crack were swollen in response to water absorption and partially blocked the crack, as can be seen in the white boxed areas. This result revealed that the WSRPs hold a potential to be applied for self-sealing cracks in mortar with the water swelling function.

### 3.3. Influence of WSRPs on the Mechanical Properties of Mortar

It is known that the use of 1% superabsorbent polymer (SAP) will results in an around 18% decrease in strength [12,30]. Similarly, a significate decrease in concrete compressive strength with increasing amount of rubber phase in the mixture can always be detected regardless the different nature, size and composition of tyre rubber [16,31]. The result shows that the compressive strength of rubberised concrete decreased 20%, 28% and 64% by adding 2%, 3% and 6% of tyre rubber in concrete [32]. In this study, the influence of incorporating 1%, 3% and 6% of WSRPs on the mechanical properties of mortar was evaluated systematically. Section 2.2 explains the specimen preparation for the mechanical tests. For each mix 3 replicate prisms were cast. 10% of WSRPs replacement has also tried, while since it detrimentally influence on the mechanical properties of mortar, so the results are taken into our system consideration in this paper.

**Figure 7:**
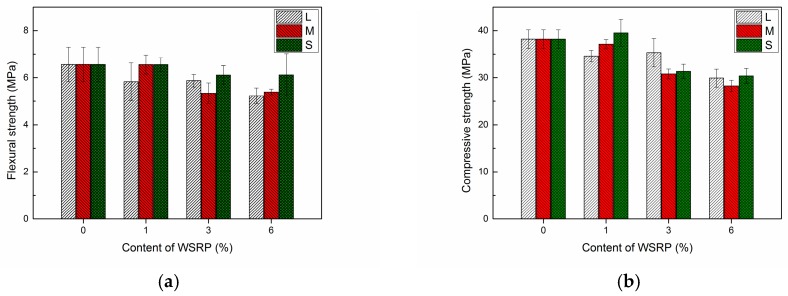
Influence of WSRPs incorporation on the (**a**) flexural strength and (**b**) compressive strength of specimens (with different additions of WSRPs, 0% (R), 1%, 3%, 6% of sand by volume percentage, respectively) at the age of 28 days of hydration.

Figure 7a shows the relationship between the flexural strength and content of WSRPs. The error bar represents the standard deviation of the test results. It is clear that the addition of WSRPs has a negative effect of the flexural strength of the specimens. For the L series specimens, the flexural strength was decreased by values ranging from 10.5 to 20% with increasing the content of WSRPs from 1 to 6 %. The flexural strength of M series specimens decreased up to 18% for those specimens containing 6% WSRP. The addition of S size WSRPs has the least influence on the flexural strength. It only decreased 6% of its flexural strength for a dosage of 6% WSRPs. As expected, the incorporation of WSRPs also had a negative effect on the compressive strength of the specimens. As shown in Figure 7b, by incorporation of 1%, 3% and 6% of WSRPs, the compressive strength for S, M and L series specimens reduced by 0–10%, 8–18% and 20–26% respectively.

**Figure 8:**
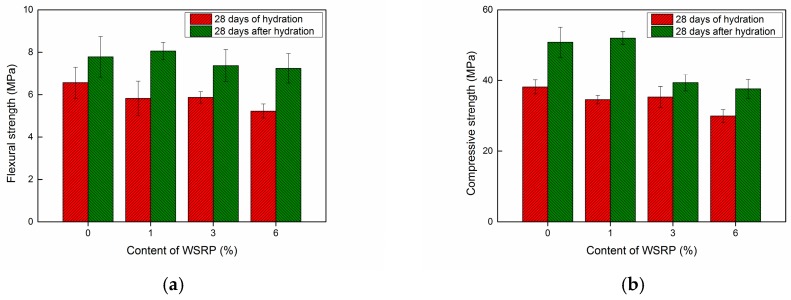
Influence of WSRPs incorporation on the (**a**) flexural strength and (**b**) compressive strength of specimens at different ages (28 days of hydration and 28 days storage at lab environment after 28 days of hydration).

As it is expected that the addition of WSRPs will have a long-term effect on the internal strength and humidity, the mechanical properties of the specimens at a later age (28 days of hydration and then 28 days stored in laboratory environment) were investigated on the L series specimens. The results are shown in Figure 8. According to the results, a significant increase was observed for both flexural and compressive strength in each series of specimens. For the flexural strength, a maximum of 38.7% increase was found for specimens containing 6% of large size WSRPs at age of 56 days compared to 28 days. Regarding the compressive strength, an obvious increase was noticed for the R and L1 specimens while only slight increase was found on L3 and L6. It can be seen that longer storage time can enhance the mechanical properties for both of the specimens with and without WSRPs. The main reason for this can be attribute to the further hydration of those unhydrated cement powder. Meanwhile, it is noteworthy that after a 28 days storage in laboratory environment, both flexural strength and compressive strength of WSRPs containing specimens were even higher or similar to the reference specimens with 0% of WSRP at 28 days of hydration. The possible reason for this enhancement can be partly attributed to the fact that, after a 28 days storage in laboratory environment, the WSRPs in specimens has less swelling pressure than the specimens which were just taken out from the fog room at 28 days of hydration, which will result in a lower stress concentration in the specimens and, therefore, a higher mechanical strength of specimens. Another explanation for this is that the absorbed water in WSRPs during the hydration process promoted a further hydration of the cement matrix by yielding their absorbed water into the surrounding cement matrix for the formation of new C–S–H crystals after 28 days of hydration. This can be deduced from the obviously slightly higher increase of flexural strength in the specimens with WSRPs than the reference specimen with 0% of WSRPs after a 28 days storage in laboratory environment. Similar describe can be found for superabsorbent polymer. While the details of further hydration function is beyond the scope of this paper and will be analyzed and described in further publications.

### 3.4. Self-Sealing Effect 

**Figure 9:**
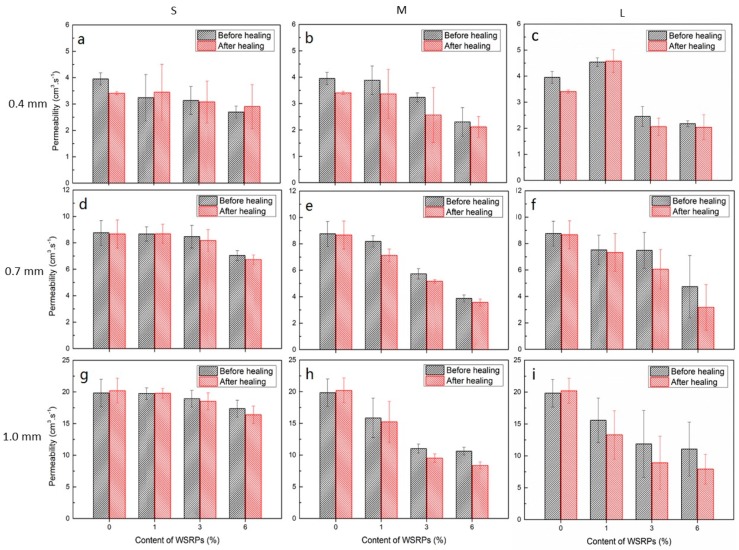
Data of permeability test of series S, M, L and B comparing the permeability at 0 day and after 7 days of water healing process in the form of 9 bar chart graphs. Graphs (**a**,**d**,**g**) show the permeability data of S series samples with different crack width (0.4, 0.7 and 1.0 mm); Graphs (**b**,**e**,**h**) show the permeability data of M series samples with different crack width (0.4, 0.7 and 1.0 mm); Graphs (**c**,**f**,**i**) show the permeability data of L series samples with different crack width (0.4, 0.7 and 1.0 mm).

The permeability of specimens before and after the healing process was measured and plotted. Figure 9 shows the data comparing the permeability of samples after 0 and 7 days of immersion with different content of WSRPs (0% (R), 1%, 3%, 6% of sand by volume percentage) and different crack width in the sample (0.4 mm, 0.7 mm and 1.0 mm).

For 0.4 mm crack width samples, the permeability decreased within the range of 13–27% with the addition of 1–6% of small size rubber filler (S series). In this particular experimental setup, reductions caused by 1% concentration of medium size (M) and large size (L) rubber fillers were negligible. However, the performance of specimens containing 3–6% of both medium (M) and large (L) size rubber fillers were more advanced compared to the small size particles (S) series with 18–42% and 38–45% respectively. Despite the permeability reductions due to the addition of WSRPs, only few percentage points of self-sealing capability can be found on most of the 0.4 mm crack width samples by comparing the permeability before and after healing process. For 0.7 mm crack width samples, the S series still show limited effect with only maximum 18% decrease of permeability. While medium series (M) and large series (L) rubber systems showed much better performance in permeability reductions, ranging of 17–56% and 16–64% consecutively for WSRPs concentration of 1–6%. Although the value of the flow rate is much high than for samples with 0.4 and 0.7 mm crack, samples with 1.0 mm crack width had a similar trend of permeability reductions. A permeability reduction of 22–58% and 23–61% can be found for the medium size (M) and large size (L) rubber systems with 1.0 mm crack width. While there is no significant decrease of permeability for small size (S) rubber system.

Moreover, a noticeable improvement could be found by comparing the permeability decreases before and after the rubber was allowed to swell after 7 days of immersion (self-sealing process). This self-sealing effect became more prominent for the samples subjected to crack widths of 0.7 mm and 1 mm as they showed relatively steeper decrease of permeability. In general, samples which were prepared with higher concentration and larger particles size seemed to show more significant further permeability reduction by the self-sealing effect of WSRPs. For 0.7 mm crack width sample, the swollen rubber for medium series (M) sample could lower the permeability to about 6–12% compared to the sample before immersion. While, the swollen rubber of large series (L) rubber could generate further reduction of about 3–20% with the rubber concentration of 1–6%. In addition, in 1.0 mm crack width samples the permeability can be further lowered in the range of 3–22% and 12–24% respectively for both medium (M) and large (L) samples.

In general, based on the results of reducing water permeability of the mortar specimens, the reduction of water permeability in this study can be attribute to two reasons. The first reason is the exist of WSRPs on the crack partly block the path of water flow. This can be reflected form the immediately decrease of permeability for those sample with WSRPs embedded, even for those “before healing” series. The second reason is the swelling effect of WSRPs further enlarge the volume of WSRPs which blocking the crack that enable the permeability decrease further. This can be reflected by the further decrease of permeability after 7 days healing. Meanwhile, the large series (L) samples performed almost similar to the medium series (M) samples for both 0.7 mm and 1 mm crack width. These occurrences can be attribute to the balance between limitations of rubber expansion and the spatial availability within the cracked sample. This means that swelling of large size particles is more difficult since the dimensions of the spaces in which it has to squeeze through are smaller in comparison. Another matter to consider is the significantly higher scatter in the data collected for the large specimen series (L) compared to the medium size samples (M) due to the lower probability of particle homogenisation. Since, in order to occupy the same volumetric ratio, less large particles are needed while more grains of small and medium size are required which causes uneven particles distribution within the mortar samples. Then it ultimately causes L series sample to have a lower probability to be homogenously present in the crack path. Therefore, although L and M size particles may seem to produce the same average permeability reduction in this study, there are chances in which the self-sealing function of L series samples may underestimated because of the inferior particle distribution.

### 3.5. Visual Assessment of the Crack Bridging Function of WSRPs

**Figure 10:**
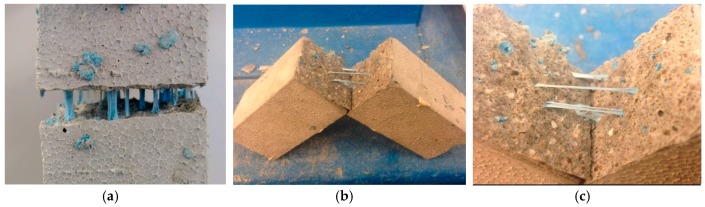
Typical images showing the crack bridging function of mortar with (**a**) 4% and (**b**,**c**) 1% of WSRPs as a replacement of sand.

The crack bridging function is a unique characteristic of WSRPs. The embedded WSRPs can provide the concrete with a bridging effect to prevent the crack from fully separating the specimen. Typical images of the crack area of the WSRPs embedded sample were examined by digital camera and are shown in Figure 10. As can be seen from the Figure 10a, after the tensile load was first imposed and then removed from the cracked sample loaded with 4% of WSRPs, the blue rubber particles become visible inside the crack. It is clear that the cracked specimen halves were kept together by the WSRPs and the specimen is not separated into two parts. This phenomenon indicates that the embedded rubber has the ability to bridge the cracked faces of the specimen. Figure 10b,c shows the internal crack surface of sample with only 1% of WSRPs. It can be seen that, even if the specimen was completely broken, the remnant of rubber particles were still binding to the mortar and the original grainy rubber was stretched into a fiber-like structure. This observation demonstrates that the rubber has a good bonding strength with mortar matrix, which corresponds well with the result of the microstructure study by ESEM in Section 3.1.

## 4. Conclusions

In this study, granulated water swelling rubber (WSRPs) was first used for reducing permeability of large cracks (width: 0.4 to 1 mm) through volume blocking and volume expansion triggered by water absorption. The XCT result shows that the WSRPs have an obvious self-sealing effect in the cracked mortar. The incorporation of MSRPs into the system lowered the compressive strength by maximum of 19% with addition of 6% of large size WSRPs, and the flexural strength by maximum of 20% with incorporation of 6% large size WSRPs. The sealing function of WSRPs in mortar was studied for cracks of 0.4 mm, 0.7 mm and 1.0 mm wide. It was found that the addition of WSRPs in mortar was able to partially lower the permeability, which was decreased even further after the samples were immersed in water for 7 days. In general, degree of permeability reductions increases with higher concentration of WSRPs. Larger particles shows higher effect of the self-sealing function. The data collected from permeability test suggests that medium (M) and large (L) particles showed more or less similar impact in permeability decrease. A maximum permeability reduction of 58% and 64% can be found for the medium size (M) and large size (L) rubber systems with WSRPs concentration of 6%. In addition, a crack bridging function of WSRPs that can bridge the cracked mortar and prevent the two halves from completely separating was discovered.

This work contributes to the search for an effective material to completely or partially decrease the permeability of cementitious materials with less influence on mechanical properties. Comparing with previous study, the novel preparation and application of WSRPs give the cement mortar, a traditional construction material, a self-sealing ability with less sacrificing the mechanical properties. However, as a new self-sealing agent, some improvements to the proposed study are still needed. Firstly, water swelling rubber particles which are particularly applicable to our application with less initial swelling at the hydration stage and with higher swelling pressure at the self-sealing period should be developed. Meanwhile it was found that the swelling capacity of WSRPs is highly influenced by the crack size and particle sizes. Therefore optimization procedures of size and concentration of WSRPs to increase the distribution probabilities and improve self-sealing performance should be developed. In addition, in preparing for practical application, further focus will be given to an accurate measurement of the potential of the crack bridging function of the WSRPs in cementitious materials.

## Figures and Tables

**Table 1:** The Grading of Standard CEN Sand.

**Square Mesh Size (mm)**	2	1.6	1	0.5	0.16	0.08
**Cumulative (%) Retained**	0	7	33	67	87	99

**Table 2:** Mix content of cement mortar.

Series	ID	Water (g)	Cement (g)	Sand (g)	WSRPs (g)	WSRPs/Sand (vol %)
S	S1	225	450	1338.9	11.1	1
	S3	225	450	1309.5	33.0	3
	S6	225	450	1285.7	64.3	6
M	M1	225	450	1338.9	11.1	1
	M3	225	450	1309.5	33.0	3
	M6	225	450	1285.7	64.3	6
L	L1	225	450	1338.9	11.1	1
	L3	225	450	1309.5	33.0	3
	L6	225	450	1285.7	64.3	6
B	B	225	450	1350	0	0
